# Inositol Pyrophosphates: Energetic, Omnipresent and Versatile Signalling Molecules

**DOI:** 10.1007/s41745-016-0011-3

**Published:** 2017-02-28

**Authors:** Akruti Shah, Shubhra Ganguli, Jayraj Sen, Rashna Bhandari

**Affiliations:** 1 Laboratory of Cell Signalling, Centre for DNA Fingerprinting and Diagnostics, Hyderabad, Telangana India; 2 Graduate Studies, Manipal University, Manipal, Karnataka India

**Keywords:** 5-Diphosphoinositol pentakisphosphate (IP_7_), Bis-diphosphoinositol tetrakisphosphate (IP_8_), Inositol hexakisphosphate (IP_6_), IP_6_ kinase (IP6K), Inositol phosphate

## Abstract

Inositol pyrophosphates (PP-IPs) are a class of energy-rich signalling molecules found in all eukaryotic cells. These are derivatives of inositol that contain one or more diphosphate (or pyrophosphate) groups in addition to monophosphates. The more abundant and best studied PP-IPs are diphosphoinositol pentakisphosphate (IP_7_) and bis-diphosphoinositol tetrakisphosphate (IP_8_). These molecules can influence protein function by two mechanisms: binding and pyrophosphorylation. The former involves the specific interaction of a particular inositol pyrophosphate with a binding site on a protein, while the latter is a unique attribute of inositol pyrophosphates, wherein the β-phosphate moiety is transferred from a PP-IP to a pre-phosphorylated serine residue in a protein to generate pyrophosphoserine. Both these events can result in changes in the target protein’s activity, localisation or its interaction with other partners. As a consequence of their ubiquitous presence in all eukaryotic organisms and all cell types examined till date, and their ability to modify protein function, PP-IPs have been found to participate in a wide range of metabolic, developmental, and signalling pathways. This review highlights many of the known functions of PP-IPs in the context of their temporal and spatial distribution in eukaryotic cells.

## Introduction

**Figure 1:**
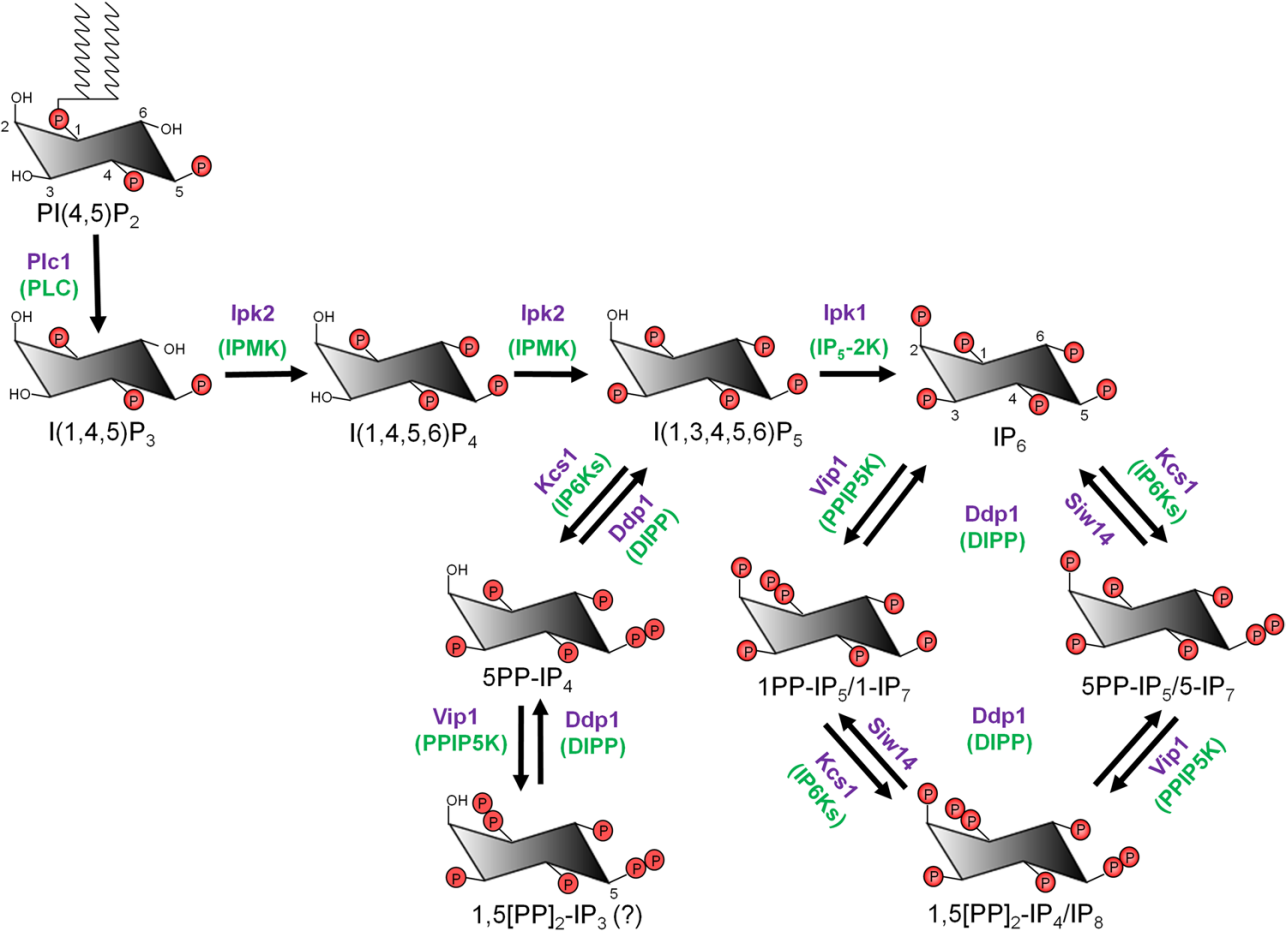
The pathway of synthesis of inositol pyrophosphates. The simplest pathway in yeast starts with the formation of IP_3_ from PI(4,5)P_2_ by the action of phospholipase C (PLC). Subsequent phosphorylation by Ipk2 (IPMK in mammals) converts IP_3_ to IP_4_ and IP_5_. Ipk1 (IP_5_-2K in mammals) converts IP_5_ to IP_6_. Kcs1 (IP6Ks in mammals) phosphorylates IP_6_ to 5PP-IP_5_ (or 5-IP_7_). Vip1 (PPIP5Ks in mammals) acts on IP_6_ to form 1PP-IP_5_ (or 1-IP_7_) and on 5-IP_7_ to form 1,5[PP]_2_-IP_4_ (or IP_8_). Kcs1 can also convert 1-IP_7_ to IP_8_. 5PP-IP_4_ and 1,5[PP]_2_-IP_3_ are synthesised from IP_5_ by the action of Kcs1 and Vip1. IP_6_ kinases prefer IP_6_ over IP_5_ due to their higher affinity towards the former.^102^ In yeast, the minor inositol pyrophosphates, 5PP-IP_4_ and 1,5[PP]_2_-IP_3_ are detected only on deletion of Ipk1.^95^ DIPP (diphosphoinositol polyphosphate phosphohydrolase), which has five isoforms in mammals and a single isoform, Ddp1, in yeast, hydrolyses diphosphate groups on IP_7_ and IP_8_ to form IP_6_, and on PP-IP_4_ and [PP]_2_-IP_3_ to form IP_5_.^2^, ^131^ Siw14, an inositol pyrophosphate phosphatase in yeast, preferentially cleaves the C5 β-phosphate on PP-IPs.^37^ The yeast enzymes are depicted in purple, and mammalian enzymes are depicted in green and are bracketed. The undetermined inositol pyrophosphate structure is represented with an interrogation mark. *Myo*-inositol contains five equatorial (parallel to the axis) and one axial (perpendicular to the axis) hydroxyl groups. Carbon atoms on the *myo*-inositol ring are numbered on the structures of PI(4,5)P_2_ and IP_6_.

*Myo*-inositol, a stereoisomer of cyclohexanehexol with one axial and five equatorial hydroxyl groups, is a component of biomolecules found in all forms of life.^1^ In eukaryotic cells, the phosphorylated derivates of *myo*-inositol include lipid phosphatidyl inositols and water-soluble inositol polyphosphates.^1^ Inositol hexakisphosphate (IP_6_), at a concentration range of 10–100 μM in yeast and animal cells and 500 μM in slime moulds, is the most abundant inositol polyphosphate in eukaryotes.^2^, ^3^ In the early 1990s, a distinct sub-class of inositol polyphosphates containing ‘high energy’ pyrophosphate groups was identified in slime mould and mammalian cells.^4^–^6^ Since then several studies have characterized these inositol pyrophosphates (PP-IPs) and their functions (for reviews see^2^^,^ 7–23). The major PP-IPs, diphosphoinositol pentakisphosphate, PP-IP_5_ (or IP_7_) and bis-diphosphoinositol tetrakisphosphate, [PP]_2_-IP_4_ (or IP_8_), are derived by the addition of phosphate groups to pre-existing monophosphates on IP_6_ (Fig. 1). IP_7_ is the most abundant PP-IP, and its concentration ranges from 0.5 to 1.3 µM in yeast and mammalian cells.^2^ IP_8_ is present at much lower levels in most organisms, ranging from undetectable to approximately 50% of IP_7_ levels in budding yeast^24^ and some mammalian cell lines.^2^, ^25^

The pathway of synthesis of inositol polyphosphates has been characterized in yeast, slime moulds, plants and animals. The simplest anabolic pathway, characterized in the yeast *Saccharomyces cerevisiae* (Fig. 1), involves the release of I(1,4,5)P_3_ from PI(4,5)P_2_ by phospholipase C (PLC), subsequent phosphorylation of IP_3_ to IP_4_ and IP_5_ by inositol polyphosphate multikinase (IPMK or Ipk2), and the conversion of IP_5_ to IP_6_ by the IP_5_ 2-kinase, Ipk1.^26^ Inositol pyrophosphates are synthesized by two classes of enzymes, IP_6_ kinases and PP-IP_5_ kinases. IP_6_ kinases, identified by the Snyder group, convert IP_6_ to 5PP-IP_5_ (also called 5-IP_7_).^27^ *S. cerevisiae* have a single IP_6_ kinase, called Kcs1, whereas mammals have three isoforms, IP6K1, IP6K2 and IP6K3. PP-IP_5_ kinases, identified independently by the York and Shears laboratories, convert 5PP-IP_5_ to 1,5[PP]_2_-IP_4_ (IP_8_).^28^, ^29^ These kinases can also synthesize an alternative form of IP_7_ (1PP-IP_5_, also called 1-IP_7_) from IP_6_.^30^, ^31^ Yeast has a single PP-IP_5_ kinase (Vip1 in *S. cerevisiae*), and mammals have two isoforms, PPIP5K1 and PPIP5K2.^29^ 1-IP_7_ is the minor IP_7_ isoform under normal conditions.^2^, ^32^ PP-IP_4_ and [PP]_2_-IP_3_ are derived from IP_5_ by the action of IP_6_ kinases and PP-IP_5_ kinases.^2^, ^33^ Diphosphoinositol polyphosphate phosphohydrolase, or DIPP (Ddp1 in *S. cerevisiae*) hydrolyzes the pyrophosphate moiety of inositol pyrophosphates, rapidly degrading IP_8_ to IP_6_ 34 (Fig. 1). Interestingly, the PPIP5Ks also possess a phosphatase domain which selectively cleaves the 1-position β-phosphate of 1-IP_7_ and IP_8_, thus targeting the products of the kinase domain.^35^, ^36^ A recent study identified another yeast phosphatase, Siw14, that specifically targets the 5-position β-phosphate of PP-IPs^37^ (Fig. 1).

**Figure 2:**
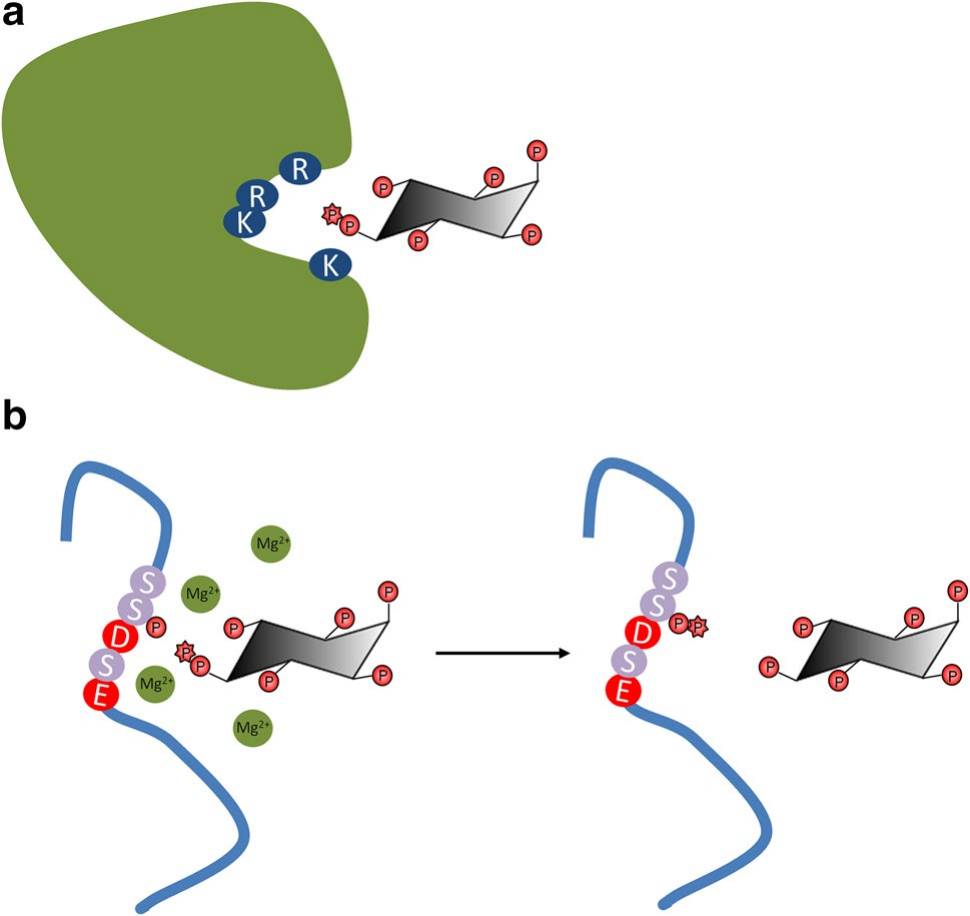
Mechanism of regulation of protein function by PP-IPs. PP-IPs modulate protein function by two mechanisms: **a** direct binding to proteins in which the positively charged binding pockets formed by Lys/Arg residues (shown in *blue*) electrostatically neutralise the high negative charge of the PP-IP molecule,^125^ and **b** protein pyrophosphorylation, which involves the non-enzymatic transfer of the β-phosphate from a PP-IP to a pre-phosphorylated Ser residue (shown in *mauve*) surrounded by Asp/Glu residues (shown in *red*).

As PP-IPs are found in all eukaryotic organisms, they display many conserved and divergent functions in yeast, plants and mammals. These small molecules participate in a wide array of cellular and organismal processes ranging from apoptosis and DNA repair to energy homeostasis and blood clotting. Inositol pyrophosphates regulate protein function via two molecular mechanisms, (a) protein binding and (b) protein pyrophosphorylation (Fig. 2). The inositol pyrophosphates 1-IP_7_ and 5-IP_7_ show isomer-specific binding to proteins to regulate their function.^38^–^41^ Conversely, any inositol pyrophosphate may act as a phosphate donor, transferring its β-phosphate to a pre-phosphorylated serine to form pyrophosphoserine, bringing about protein pyrophosphorylation^42^, ^43^ (Fig. 2). Pyrophosphorylation is an enzyme-independent reaction, requiring only the inositol pyrophosphate donor, the pre-phosphorylated protein acceptor, and divalent cations such as Mg^2+^. The acceptor serines are pre-phosphorylated by a protein kinase, usually CK1 or CK2, and occur in acidic serine sequence motifs, i.e. one or more Ser with neighbouring Glu/Asp residues. IP_7_-mediated pyrophosphorylation of specific proteins can regulate glycolysis and rRNA synthesis in yeast,^44^, ^45^ and viral particle release and dynein motor driven retrograde trafficking in mammalian cells.^46^, ^47^ Specific serine phosphatases such as PP1 and PP2C cannot remove the pyrophosphate group,^42^, ^48^ but alkaline phosphatase can depyrophosphorylate serine,^48^ suggesting that pyrophosphorylation is a reversible modification with many potential roles in cell signalling.

## Temporal Regulation of Inositol Pyrophosphates

### Detection and Measurement of PP-IPs

The biochemical determination of PP-IP levels presents several technical challenges, and till date there are no antibody-based or colourimetry-based assays to detect these molecules. Most measurements have relied on monitoring radiolabelled [^3^H]inositol or [^32^P]phosphate after strong anion exchange HPLC-based resolution to separate individual inositol phosphates. The Mayr laboratory developed a post-HPLC column derivatization method that allows spectrophotometric detection of unlabelled inositol polyphosphates,^49^ and was also able to resolve 1-IP_7_ and 5-IP_7_.^30^ A new method reported recently employs a monosaccharide binding resin (CarboPac™) to resolve 1-IP_7_ and 5-IP_7_ and has estimated that 1-IP_7_ constitutes less than 2% of total IP_7_ in a human colon cancer cell line.^32^ All HPLC-based resolution methods however require deproteinization of the sample using perchloric acid or trichloroacetic acid, and employ acidic buffers to elute inositol phosphates from chromatography columns. These acidic conditions lead to hydrolysis of the β-phosphate moiety of PP-IPs, and their likely underestimation in cells and tissues.^50^ A simple method developed by the Saiardi laboratory employing high-percentage polyacrylamide gels to resolve IP_6_, IP_7_, and IP_8_ avoids the use of low pH buffers, thereby improving the efficiency and ease of PP-IP detection. The sensitivity of PP-IP detection was also improved by staining gels with the fluorescent dye 4′,6-diamidino-2-phenylindole (DAPI),^50^ and by using titanium oxide (TiO_2_) beads to enrich for these phosphate containing molecules in cell and tissue extracts.^25^ These newer methods have suggested that earlier estimates of PP-IP levels may be off the mark.^3^ Nevertheless, while studies over the past twenty years employing HPLC methods may have underestimated the absolute levels of PP-IPs, the information on an increase or decrease in the concentration of these molecules, or changes in enzyme activity, is likely to be accurate. This section therefore presents a summary of our current knowledge of the temporal changes in PP-IP levels under different conditions.

While classical second messenger molecules like cAMP display acute changes in response to extracellular signals, most measurements suggest that PP-IP levels change only marginally under certain conditions. The levels of PP-IPs are tightly regulated and these molecules display a high turnover rate, with studies showing that the IP_7_ pool can turn over ten times every 40 min,^51^ suggesting that pathways for the synthesis and utilisation of IP_7_ are constantly active in the cell. Several studies show that IP_7_ or IP_8_ levels vary in response to physiological stresses, during different phases of the cell cycle, and over the course of development and ageing.

### PP-IPs Fluctuate in Response to Stress

IP_7_ and IP_8_ are involved in the cell’s response to different physiological stresses in mammals, yeast, and plants. In mammalian cells, hyper-osmotic stress can trigger an acute 25-fold increase in the level of IP_8_,^52^ and thermal stress can increase IP_8_ levels 3–4 fold.^53^ PPIP5K1, one of the mammalian enzymes responsible for IP_8_ synthesis from 5-IP_7_, is activated fourfold upon osmotic stress.^29^ Initial studies using protein kinase inhibitors suggested that the ERK/MEK kinase pathway was responsible for these changes in IP_8_,^52^, ^53^ but later data revealed that these kinase inhibitors actually acted via off-target effects on the cellular AMP/ATP ratio^54^ (discussed later). Exposure of cells to apoptosis inducing drugs such as cisplatin or staurosporine has also been shown to elevate the levels of IP_7_ and to a lesser extent IP_8_ 55 (discussed in detail later). Interestingly, exposure of neutrophils to nicotine or cigarette-smoke extract decreases IP_7_ levels, suggesting that IP_7_ plays a role in the pathogenesis of tobacco-induced chronic obstructive pulmonary disease.^56^

In budding yeast, the effect of phosphate starvation on inositol pyrophosphate levels has yielded conflicting results. One study observed an increase in cellular IP_7_ concentration during limiting extracellular phosphate levels,^57^ whereas others revealed a decrease in IP_7_ levels following phosphate starvation.^58^, ^59^ PP-IP levels in yeast decrease rapidly upon treatment with hydrogen peroxide, as a consequence of inhibition of Kcs1 enzyme activity via oxidation of a cysteine residue.^24^ Yeast lacking Kcs1 demonstrate reduced cell death upon exposure to H_2_O_2_ 24 but are sensitive to several other stresses, including osmotic and thermal stresses.^60^ Yeast carrying deletions for both *kcs1* and *vip1* genes have no PP-IPs and show no changes in transcription in response to osmotic, heat or oxidative stress, suggesting that PP-IPs are required for a cell to adapt in order to survive these stresses.^61^

Plant seeds are the most abundant source of IP_6_ (also known as phytic acid), which serves as a store for phosphorus, inositol, and cations required by the seedling during germination. Recently, two groups independently demonstrated the presence of IP_7_ and IP_8_ in plant seeds and vegetative tissues.^62^, ^63^ These studies also identified two Vip1 orthologues in Arabidopsis that catalyse the formation of IP_8_ from IP_7_. Although no IP6K orthologue has been identified in plants, it was speculated that an unknown enzyme activity is responsible for plant IP_7_ synthesis.^63^ It is also likely that the IP_7_ and IP_8_ isomers found in plants are different from those occurring in yeast and mammals.^63^ The plant stress hormone abscisic acid was shown to cause a twofold increase in the levels of both IP_7_ and IP_8_, whereas treatment with the plant defence hormone jasmonate

**Jasmonate**: A lipid based hormone that regulates plant growth and is required for plant defence responses against herbivores and environmental stress

led to a sustained twofold increase in IP_8_ but did not affect IP_7_ levels.^63^ Further probing the specific function of IP_8_ in jasmonate signalling, this study suggested that IP_8_ binds the F-box protein COI1 which is part of an E3 ligase complex responsible for proteasomal degradation of the transcriptional repressor protein JAZ. The combined binding of jasmonate and IP_8_ to the COI1-JAZ jasmonate coreceptor complex facilitates JAZ degradation, thereby permitting the expression of jasmonate responsive genes involved in plant defences against insect herbivores and fungal pathogens.

### PP-IP Changes During the Cell Cycle

PP-IP levels have also been shown to fluctuate during the cell cycle in both yeast and mammals. *S. cerevisiae* cells can be arrested in the G1 phase of the cell cycle by treatment with the α-factor mating pheromone.^64^ IP_7_ and IP_8_ levels increase by approximately twofold between 30 to 60 min after release from this arrest, in the time that corresponds to synchronised entry of the cells into the S phase.^65^ The levels of the PP-IPs decrease again during the G2/M phase. The activity of Kcs1 was shown to mirror these changes in IP_7_ and IP_8_ levels, suggesting that signalling pathways operating during the cell cycle may post-translationally modify Kcs1 to regulate its activity.^65^ Mammalian cells can be arrested in the G_0_ phase of the cell cycle by allowing them to grow to confluence, and they can be arrested in early mitosis by treatment with nocodazole, which interferes with microtubule polymerization and prevents the formation of the mitotic spindle.^66^ When rat mammary tumour cells were synchronised by both these methods, IP_7_ was found to be twice as high in the G1 phase as compared with the other phases of the cell cycle where the basal levels of this PP-IP are approximately 0.6 µM.^67^ The functional significance of these changes in PP-IP levels during the cell cycle is still unclear, but it is likely that one or more cell cycle modulating factors may be regulated by IP_7_ binding or pyrophosphorylation.

### PP-IPs in Development and Ageing

Unlike most other eukaryotes, the slime moulds *Dictyostelium discoideum* and *Polysphondylium pallidum* display an abundance of PP-IPs, and are also the only documented organisms in which IP_8_ is more abundant than IP_7_.^3^, ^68^ Recent measurements using TiO_2_ beads and polyacrylamide gel electrophoresis estimate that vegetative stage *D. discoideum* contain IP_7_ at 60 μM and IP_8_ at 180 μM,^3^ and that IP_8_ levels go up approximately threefold during starvation induced development, when these amoebae aggregate, form a multicellular “slug”, and eventually develop into a fruiting body. The chemoattractant cAMP released by *D. discoideum* during starvation has been shown to lead to a rapid and sustained three- to fourfold increase in IP_7_ and IP_8_ levels.^69^ IP_7_ competes with the lipid inositide PI(3,4,5)P_3_ to bind the PH domain of the protein Crac, and interferes with Crac translocation to the plasma membrane. As Crac translocation is required for cAMP-dependent chemotaxis

**Chemotaxis**: Phenomenon of movement of a cell or an organism in response to a chemical stimulus.,

it was postulated that IP_7_ is a negative regulator of chemotaxis, modulating the sensitivity of cells to cAMP stimulation.

IP_7_ levels have been shown to increase in mice as they age.^39^ While both IP_6_ and IP_7_ are higher in hepatocytes derived from 10-month-old as compared with 2-month-old mice, the IP_7_/IP_6_ ratio increases more than twofold in older mice, and correlates with a decrease in insulin sensitivity. Like *D. discoideum* Crac, the PH domain of mammalian AKT can bind IP_7_, which competes with PI(3,4,5)P_3_ binding to inhibit membrane translocation and activation of AKT in response to insulin. *Ip6k1* ^−/−^ mice with reduced levels of IP_7_ and upregulated AKT signalling are thus more insulin sensitive as they age. Another study demonstrated increased IP_7_ production in ageing bone marrow derived mesenchymal stem cells as compared to young cells.^70^ Consequently, there is decreased AKT phosphorylation and activation in older cells, leading to increased sensitivity to hypoxic injury with age.

## The Functions of PP-IPs in Different Cellular Compartments

Technical limitations imposed by currently available methods for the detection of PP-IPs have translated into the lack of any convincing data on the subcellular compartmentalisation of PP-IPs. However, there are several studies on the tissue distribution and subcellular localisation of the IP6Ks and PPIP5Ks, which suggest that IP_7_ and IP_8_ are likely to be found in a variety of tissues and in all cell compartments. In *S. cerevisiae*, both PP-IP kinases, Kcs1 and Vip1 are located predominantly in the cytoplasm (http://yeastgfp.yeastgenome.org/), but several studies have revealed important functions for PP-IPs in various subcellular compartments including the nucleus and vacuoles. In mice, *Ip6k1* and *Ip6k2* mRNA are expressed at varying levels in all tissues,^27^ whereas IP6K3 is highly expressed in the cerebellum.^71^, ^72^ *Ip6k1* mRNA shows highest expression in testes,^27^ correlating with spermatogenesis failure observed in *Ip6k1* knockout mice.^73^ Although protein overexpression studies show IP6K1 located in the cytoplasm and nucleus, IP6K2 mainly in the nucleus, and IP6K3 localised predominantly in the cytoplasm,^71^ such analyses are plagued with anomalies arising from high levels of unregulated protein expression, and often do not reflect the behaviour of the endogenous protein. *Ppip5k1* mRNA is expressed in many human tissues, with greater abundance observed in skeletal muscle, heart and brain.^74^ Overexpressed PPIP5K1 and PPIP5K2 localise mainly to the cytoplasm,^29^, ^74^ but PPIP5K2 has a nuclear localisation signal that is absent in PPIP5K1, enabling its translocation to the nucleus in a phosphorylation-dependent manner.^75^ Both the PPIP5K isoforms possess a lipid inositide binding domain distinct from their kinase domains, and agonist-stimulated production of PI(3,4,5)P_3_ can lead to translocation of PPIP5K1 from the cytoplasm to the plasma membrane.^41^, ^76^

**Figure 3:**
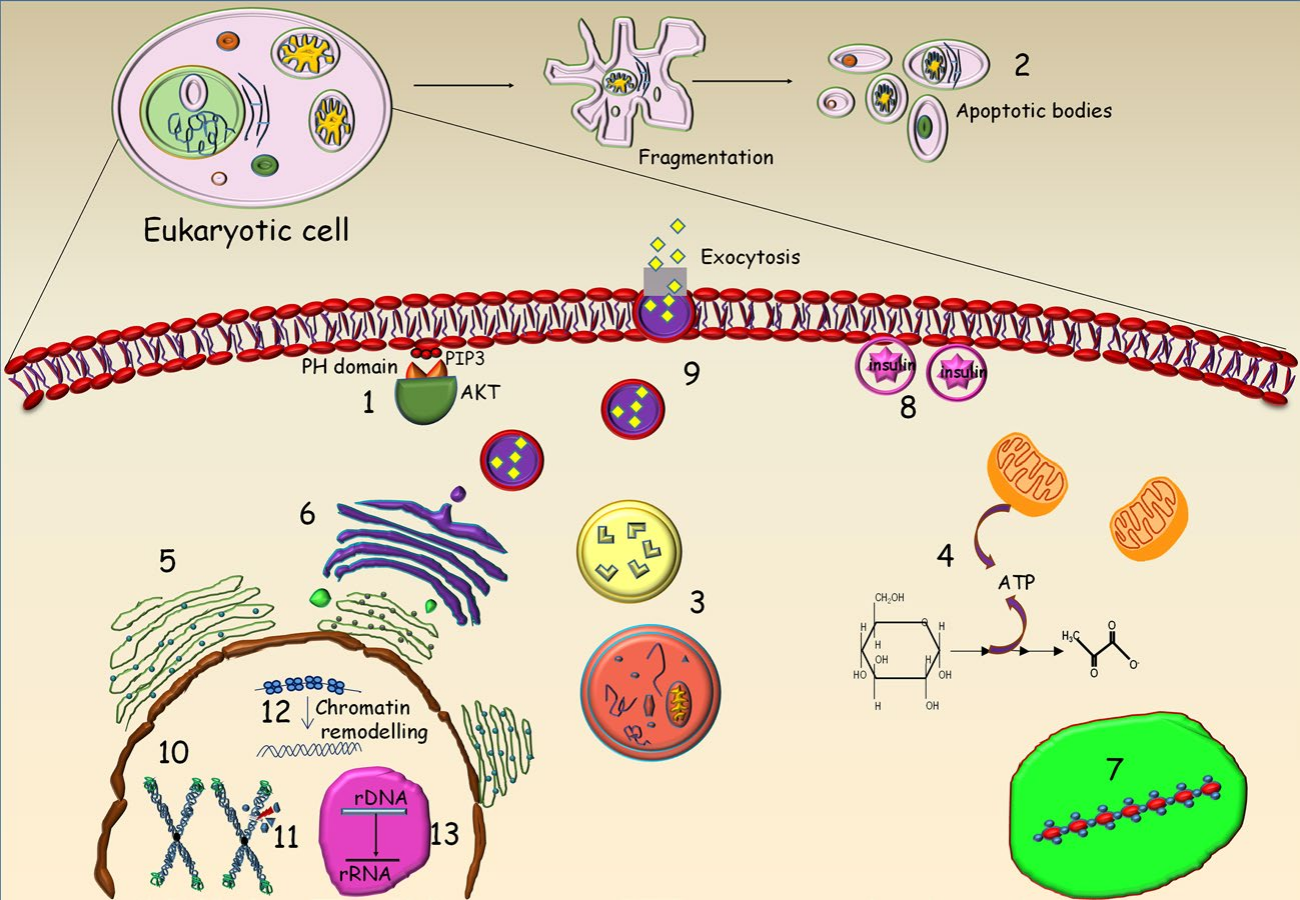
Representation of cellular functions of inositol pyrophosphates in an eukaryotic cell. *1* IP_7_ physiologically inhibits AKT signalling by competitively binding to the PH domain of AKT and thus preventing it from binding to PI(3,4,5)P_3_ (PIP3). IP_7_ and IP6K2 promote, *2* apoptosis and *3* formation of autophagosomes. *4* PP-IPs regulate the cellular levels of ATP by their action on the glycolysis pathway and mitochondrial membrane potential. *5* IP_7_ is responsible for maintaining endoplasmic reticulum morphology in yeast cells. *6* IP_7_-mediated pyrophosphorylation regulates dynein binding to membranes and thereby influences Golgi morphology. *7* PP-IPs positively regulate the synthesis of polyphosphates in yeast vacuoles. *8* In pancreatic β cells, IP_7_ upregulates insulin secretion by increasing the readily releasable pool of insulin granules docked at the plasma membrane. *9* IP_7_ inhibits the synaptic exocytotic pathway in neurons. In the nucleus, PP-IPs are responsible for *10* telomere length maintenance in yeast, *11* DNA repair via the homologous recombination (HR) and nucleotide excision repair (NER) pathways, and *12* epigenetic modifications that influence chromatin remodelling to control global transcription. *13* In yeast, IP_7_ influences ribosome biogenesis by regulating nucleolar rDNA transcription.

As suggested by the ubiquitous expression and localisation of the kinases responsible for their synthesis, PP-IPs have been shown to participate in a myriad functions in many different tissues and subcellular compartments. This section describes several functions of these small molecules in different locations within a cell (Fig. 3).

### Plasma Membrane

One of the most intriguing yet controversial functions of IP_7_ is its ability to compete with the lipid inositide PI(3,4,5)P_3_ for binding to PH domains in specific proteins, and thereby displacing these proteins from the plasma membrane. The first demonstration that IP_7_ can compete with PI(3,4,5)P_3_ to bind a PH domain was in Dictyostelium^69^ (discussed earlier). This study also demonstrated that IP_7_ binds the PH domain of the mammalian kinase AKT with an affinity comparable to I(1,3,4,5)P_4_, which mimics the PI(3,4,5)P_3_ head group. A subsequent study went on to show that the IP_7_-AKT interaction is physiologically relevant, as *Ip6k1* ^−/−^ mice show higher AKT activity, leading to increased insulin sensitivity and reduced weight gain in response to a high-fat diet.^39^ It has also been shown that enhanced PI(3,4,5)P_3_-dependent AKT activation in neutrophils derived from *Ip6k1* ^−/−^ mice leads to greater phagocytic and bactericidal ability in these cells.^40^ Although one report showed that IP_7_ is not able to displace PI(3,4,5)P_3_ from AKT in an in vitro binding assay,^77^ it was later demonstrated that non-hydrolysable analogues of 5-IP_7_ or 1-IP_7_ can inhibit AKT activation even in the presence of PI(3,4,5)P_3_.^78^ A recent study revealed that chemically synthesized 5-IP_7_ released into mammalian cells is able to induce translocation of AKT from the plasma membrane to the cytoplasm.^79^ It has been shown that AKT and other PH domains bind 5-IP_7_ and IP_6_ with higher affinity as compared with 1-IP_7_ or IP_8_.^41^ This study also suggested that PI(3,4,5)P_3_ dependent recruitment of PPIP5K1 to the plasma membrane would deplete subplasmalemmal 5-IP_7_ and IP_6_ by using them as substrates, thus providing positive feedback to PI(3,4,5)P_3_ binding by PH domains at the plasma membrane. Although the structural conundrum of how the AKT PH domain which specifically binds PI(3,4,5)P_3_ over PI(4,5)P_2_ can bind 5-IP_7_ better than IP_6_ remains unresolved, it is likely that binding of specific PP-IPs to different PH domains is a conserved mechanism by which these molecules regulate signalling at the plasma membrane.

### Cytoplasm

Studies in yeast and mammalian cells have revealed many functions for PP-IPs in the cytoplasm, including key roles in energy metabolism, apoptosis and autophagy.

#### Energy Metabolism

PP-IPs have been described as ‘energy sensors’ and ‘metabolic messengers’. This is because PP-IP levels can both reflect and regulate the cellular levels of ATP. The IP6Ks have a very low affinity for ATP, with a Km of approximately 1 mM, which lies within the range of cellular ATP concentrations. Consequently, fluctuations in ATP levels correlate with changes in the intracellular concentration of IP_7_. Lowering the cellular ATP concentration by treatment with sodium azide, oligomycin, or certain kinase inhibitors has been shown to significantly reduce the levels of PP-IPs.^54^, ^80^ Interestingly, at a low ATP/ADP ratio, IP6Ks can switch to being ADP phosphotransferases, transferring the 1-phosphate from IP_6_ to ADP to generate I(2,3,4,5,6)P_5_,^81^ depleting cytosolic IP_6_ and perhaps further lowering IP_7_ synthesis. It is possible that the dual enzymatic activity of IP6Ks allows them to function as cellular adenylate energy sensors, converting IP_6_ to IP_7_ or IP_5_ under high or low energy conditions respectively, so that these products may transduce information on the cellular energy status to regulate different metabolic and signalling pathways.

PP-IPs in turn affect ATP levels by regulating glycolysis. *S. cerevisiae* lacking Kcs1 have a higher cellular ATP concentration than wild-type yeast.^44^ It was shown that IP_7_ produced by Kcs1 pyrophosphorylates the major glycolytic transcription factor Gcr1, which is known to interact with Gcr2. In *kcs1*Δ cells, reduced pyrophosphorylation of Gcr1 increases its interaction with Gcr2, thereby increasing glycolytic flux

**Glycolytic flux**: Rate at which molecules proceed through the glycolysis pathway..

In mammals, IP6K1 has been shown to influence fat accumulation by regulating adipocyte energy metabolism.^82^ The AMP-activated protein kinase (AMPK) signalling pathway augments the transformation of white adipose tissue to beige, enhancing energy expenditure in the form of heat, and correlating inversely with type II diabetes and fat-induced obesity. IP_6_ causes stimulatory phosphorylation of AMPK, thereby activating the pathway that leads to browning of white adipose tissue. Specific deletion of IP6K1 in mouse adipocytes led to increased thermogenic energy expenditure in these cells, presumably due to increased availability of IP_6_. It was suggested that this phenomenon, coupled with increased insulin sensitivity due to AKT activation (described earlier), leads to reduced weight gain when *Ip6k1* ^−*/*−^ mice are provided a high-fat diet.

#### Apoptosis

5-IP_7_ produced by IP6K2 has been shown to upregulate apoptotic signalling pathways in many mammalian cells and tissues. Overexpression of either of the three IP6K isoforms in various cancer cell lines under normal as well as stress conditions leads to increased cell death.^55^, ^83^, ^84^ However, siRNA mediated depletion of only IP6K2 and not IP6K1 or IP6K3 abrogated cell death, suggesting that IP6K2 is the only isoform that is physiologically capable of inducing apoptosis.^55^, ^85^ Work by the Lindner group revealed that overexpressed IP6K2 in untreated cancerous or non-cancerous cells is mainly cytoplasmic and upon apoptotic induction it translocates to the nucleus.^84^ IP6K2 has a nuclear localization signal, the removal of which keeps the protein in the cytoplasm, lowering its pro-apoptotic effect. The Snyder group showed that overexpressed IP6K2 translocates to the mitochondria upon cytotoxic stress and co-localizes with the pro-apoptotic protein Bax.^55^ Overexpression of catalytically inactive IP6K2 did not promote stress-induced apoptosis, indicating that the pro-apoptotic function of IP6K2 relies on IP_7_ synthesis. Indeed, the levels of IP_7_ were found to be higher in cells undergoing cytotoxic stress as compared with untreated cells. IP6K2 has been shown to promote apoptosis by acting on several signalling pathways. IP_7_ binding to the PH domain of AKT inhibits AKT-dependent prosurvival signalling,^69^ indirectly enhancing apoptosis.^55^, ^86^ IP6K2 also influences apoptotic signalling via the transcription factor p53, which is known to activate both pro- and anti-apoptotic genes. Kinase activity independent direct binding of IP6K2 to p53 augments apoptosis by downregulating expression of p21, which is responsible for stressed cells choosing cell cycle arrest instead of apoptosis.^87^ IP6K2 also binds TTI1, a protein that forms part of the TTT co-chaperone complex that stabilises the protein kinases DNAPKcs and ATM. 88 IP_7_ synthesized by IP6K2 binds the protein kinase CK2 and promotes its phosphorylation of the TTT complex, which in turn stabilises DNAPKcs and ATM, leading to increased phosphorylation of p53 and activation of cell death. In another study, IP6K2 binding to TRAF2 (TNF receptor associated factor) prevents TAK1 (transforming growth factor β-activated kinase) phosphorylation and subsequent NFκB activation, sensitizing cells to TNF-α induced apoptosis.^89^ The heat shock protein HSP90 has been shown to bind IP6K2 and inhibit its catalytic activity, thereby protecting cells from apoptosis and augmenting cell survival.^90^ The importance of IP6K2 in promoting apoptosis is also reflected in phenotypes observed in *Ip6k2* knockout mice.^91^ Chronic exposure to the UV mimetic carcinogen 4-nitroquinoline 1-oxide (4NQO) led to a fourfold increased incidence of invasive squamous cell carcinoma of the oral cavity and oesophagus in *Ip6k2* ^−/−^ mice as compared with their wild-type littermates. This was attributed at least in part to resistance to cell death in tissues lacking IP6K2. Whole genome expression profiling of tissues from *Ip6k2* ^−/−^ as compared with *Ip6k2* ^+/+^ mice showed upregulation of certain oncogenes and downregulation of tumour suppressor genes, suggesting that other pathways regulated by IP6K2 also impinge upon survival versus death signals to influence carcinogenesis.

**Apoptosis**: Regulated or programmed cell death seen in multicellular organisms.

#### Autophagy

IP6Ks in yeast and mammals have also been shown to participate in autophagy, a process by which cytoplasmic proteins and organelles are degraded. Autophagy is initiated with the formation of the phagophore, a double membrane that begins to engulf macromolecules and organelles in the cytoplasm.^92^ The phagophore membrane closes to form an autophagosome, which fuses with a lysosome, leading to degradation and recycling of the engulfed components. Budding yeast deficient in Kcs1 display undetectable levels of PP-IPs and exhibit a reduction in the number and size of autophagosomes formed upon nitrogen starvation.^93^ *kcs1*Δ yeast show mislocalisation of phagophore assembly sites to the plasma membrane, suggesting that Kcs1 is required for normal autophagosome formation. Overexpression of IP6Ks in mammalian cells led to an increase in the number of stress-induced autophagosomes as compared with control cells, and reduced expression of IP6Ks using RNA interference suppressed autophagosome formation.^94^ Expression of an inactive IP6K had no effect, revealing that autophagosome formation is IP_7_ dependent. The evolutionarily conserved protein kinase mTOR is a master regulator of cell growth and metabolism, and negatively regulates autophagy. In human cells, IP6K expression levels showed an inverse correlation with mTOR activity, suggesting that IP_7_ may promote autophagy via the mTOR signalling pathway.^94^

### Membrane Bound Organelles

PP-IPs are not membrane permeant, and there is little evidence to suggest that PP-IP synthesising enzymes are localised within membrane-bound organelles in mammalian cells. However, changes in intracellular PP-IP levels have been shown to impact normal function in several membrane bound organelles, suggesting that these molecules may be synthesized within these organelles, may act on the cytoplasmic face of the membrane, or may be transported across membranes by yet unknown mechanisms.

#### Mitochondria

Yeast devoid of Kcs1 and mouse embryonic fibroblasts (MEFs) lacking IP6K1 have dysfunctional mitochondria, which display a significantly lower oxygen consumption as compared with their wild-type counterparts.^44^ Without IP6K1, mitochondria are unable to generate the membrane potential required for ATP production, and are also deficient in the respiratory chain protein cytochrome c oxidase. Despite these mitochondrial defects, *kcs1*Δ yeast show higher ATP levels because of increased glycolytic flux (discussed earlier). However, *kcs1*Δ yeast shows a decreased growth rate as compared with wild-type cells, which could be partly attributed to defects in the bio-synthesis of major macromolecules like fatty acids and nucleotides that depend on enzymes of the mitochondria.^44^

#### Endoplasmic Reticulum

The endoplasmic reticulum (ER) is generally nondescript and parallel to the plasma membrane in wild-type budding yeast, whereas in *kcs1*Δ cells, it appears hypertrophied and perpendicular to the plasma membrane.^95^ This suggests that PP-IPs have a role to play in ER function. However, there was no apparent defect in the secretion of newly synthesised proteins into the medium by *kcs1*Δ yeast, suggesting that ER functions remain largely unaffected in the absence of significant levels of PP-IPs. There are no studies examining whether PP-IPs influence ER structure or function in mammalian cells.

#### Golgi

Our recent studies show that *Ip6k1* ^−*/*−^ MEFs display a fragmented Golgi morphology when compared with *Ip6k1* ^+*/*+^ MEFs.^47^ This phenotype was restored by the expression of catalytically active IP6K1 but not by the inactive protein, suggesting that PP-IPs are essential to maintain Golgi morphology. The pericentriolar position of the Golgi apparatus in mammalian cells is dependent on the cytoplasmic motor protein dynein, and the Golgi appears fragmented if dynein function is compromised.^96^ Further analysis showed that PP-IPs regulate dynein binding to membranes, and thereby influence Golgi morphology (discussed later).

#### Lysosomes and Related Organelles

The number and distribution of lysosomes marked by the protein LAMP2 (lysosome associated membrane protein 2) appears unaltered in *Ip6k1* ^−*/*−^ MEFs (Chanduri and Bhandari, unpublished). However, PP-IPs have an important function in yeast vacuoles, which are lysosome-like organelles.^97^ Yeast vacuoles are the site of synthesis and storage of inorganic polyphosphates (polyP), which are linear chains of orthophosphate moieties linked via phosphoanhydride bonds.^98^ PolyP are found in all life forms and are involved in diverse physiological functions like energy metabolism, transcription regulation, and blood clotting.^98^ An intriguing link was established between PP-IPs and polyP, when it was observed that *kcs1*Δ yeast also have substantially reduced levels of polyP.^99^ Our work showed that the link between PP-IPs and polyP levels is also conserved in mammals.^100^ *Ip6k1* ^−*/*−^ mice have reduced levels of polyP in platelet dense granules, which are lysosome-related organelles. Consequently, these mice display delayed clotting time and altered clot architecture, making them less susceptible to thromboembolism

**Thromboembolism**: Obstruction of a blood vessel by a blood clot..

Elegant structural and biochemical studies from the Mayer laboratory uncovered the molecular link between PP-IPs and polyP. The first study identified that the yeast vacuolar transport chaperone (VTC) complex generates polyP from ATP, with the subunit VTC4 responsible for enzyme activity.^101^ A recent study determined that multiple subunits of the VTC complex, including VTC4, contain an SPX domain, which has a positively charged surface that can specifically bind inositol polyphosphates.^59^ Binding of 5-IP_7_ to this domain enhances VTC-dependent polyP synthesis in isolated vacuoles, suggesting that PP-IPs modulate polyP synthesis by allosteric regulation of the polyP polymerase. As the levels of 5-IP_7_ have been shown to decrease upon phosphate starvation in yeast,^58^, ^59^ it was speculated that PP-IPs may act as sensors of inorganic phosphate (Pi) levels in the cytoplasm and communicate Pi fluctuations to SPX domains, which then interact with various proteins to regulate Pi uptake, transport and storage.^59^

#### Vesicles

Several studies have investigated the role of PP-IPs in vesicle trafficking processes. Budding yeast lacking Kcs1 show abnormally small and fragmented vacuoles,^102^ which reflect a defect in the endocytosis pathway.^95^ In these cells, the endosomes formed upon endocytosis fail to fuse with vacuoles and accumulate as large multilamellar endosomal intermediates. The exact mechanism by which PP-IPs regulate endocytosis remains unclear. PP-IPs synthesized by IP6K1 are essential to ensure normal insulin secretion in mammals. *Ip6k1* ^−*/*−^ mice have lower serum insulin levels as compared with their wild-type littermates,^73^ and in pancreatic β cells, 5-IP_7_ synthesized by IP6K1 upregulates insulin secretion.^103^ 5-IP_7_ increases the readily releasable pool of insulin containing granules which are docked at the plasma membrane, ready for the first phase of exocytosis upon glucose uptake by β cells.^103^ IP6K1 can also influence synaptic vesicle exocytosis. IP6K1 acts independent of its catalytic activity to bind GRAB, a guanine-nucleotide exchange factor (GEF) for the small G-protein Rab3A, which is a negative regulator of synaptic vesicle exocytosis.^104^ IP6K1 binding to GRAB inhibits the activity of Rab3A to augment neurotransmitter release from synaptic vesicles. In contrast, a recent study showed that 5-IP_7_ synthesized by IP6K1 can inhibit the synaptic exocytotic pathway by interacting with synaptotagmin, which is a calcium-sensing protein in the synaptic vesicle membrane and promotes vesicle fusion.^105^ 5-IP_7_ (but not 1-IP_7_ or IP_6_) binds and restrains synaptotagmin in a fusion-incompetent conformation to inhibit neurotransmitter release. These contrasting studies suggest that PP-IPs and their kinases can regulate exocytosis via multiple non-overlapping pathways in different cell types and tissues.

In addition to regulating vesicle fusion events, PP-IPs have also been shown to influence vesicle transport along microtubules. In the first demonstration of how serine pyrophosphorylation can regulate cellular processes, the Saiardi laboratory showed that pyrophosphorylation of the β subunit of the adaptor protein complex AP3 can regulate the release of HIV-1 virus like particles from mammalian cells.^46^ 5-IP_7_-mediated pyrophosphorylation of AP3B1 was shown to inhibit its interaction with the microtubule plus end-directed kinesin motor protein Kif3A, thereby lowering kinesin-dependent intracellular trafficking of HIV1 virus like-particles. Recent work from our laboratory has shown that 5-IP_7_-mediated protein pyrophosphorylation can also regulate dynein-motor driven microtubule minus-end directed vesicle trafficking.^47^ We found that pyrophosphorylation of Ser51 on the dynein-intermediate chain (IC) is important for its interaction with the p150^Glued^ subunit of the dynactin complex, and is required to recruit dynein to vesicles. *Ip6k1* ^−/−^ MEFs have reduced interaction between dynein IC and p150^Glued^, and as a consequence have defects in several dynein-dependent trafficking processes, including endosomal sorting of transferrin and Golgi morphology.

### Actin Cytoskeleton

The role of PP-IPs in actin cytoskeleton regulation was first identified by studies in the fission yeast, *Schizosaccharomyces pombe*, which possesses a Vip1 orthologue named Asp1.^28^ *Asp* (*a*rp, *s*op, *p*rofilin interactor) was originally identified as a high-copy suppressor of a mutation in Arp3, which is part of the actin-related protein complex Arp2/3, an essential component of the actin cytoskeleton in yeast.^106^ *asp1*Δ yeast exhibit temperature sensitive morphological defects and defects in polarised growth due to a disorganised actin cytoskeleton. Later, when Vip1 and Asp1 were found to possess inositol pyrophosphate synthesis activity, it was observed that the kinase activity of Asp1 was essential to maintain the normal rod-shaped morphology of fission yeast.^28^

Remodelling of the actin cytoskeleton is essential for cell migration and invasion associated with carcinogenesis. Recent studies have shed light on the role of IP_7_ in the promotion of tumour growth and metastasis

**Metastasis**: Spread of cancer cells from one part of the body to another part via the blood or lymphatic system.

using in vitro and in vivo approaches.^107^, ^108^ The Snyder group showed that gene deletion of *Ip6k2* in HCT116 human colorectal cancer cells and *Ip6k2* knockdown in breast and lung cancer cell lines led to a reduction in focal adhesion kinase (FAK) phosphorylation, correlating with reduced cell spreading and cell–matrix adhesion.^107^ Epithelial to mesenchymal transition properties including cell migration and invasion were also significantly reduced in these cells. Subcutaneous xenograft of *Ip6k2* knockout HCT116 cells in immune-compromised mice led to tumours that were smaller in size as compared with those formed by parent HCT116 cells. This study also worked out the molecular mechanism underlying the influence of IP6K2 on actin cytoskeleton remodelling. Liver Kinase B1 (LKB1), which suppresses FAK phosphorylation dependent migration, invasion and metastasis, is localised in the cytosol and is activated upon phosphorylation by PKC-zeta. IP6K2 binds LKB1 and generates IP_7_ which through an unknown mechanism leads to reduced PKC-zeta dependent LKB1 phosphorylation, causing its nuclear sequestration and inactivation, thus increasing FAK activity to facilitate actin remodelling. Recent work from our laboratory showed that IP6K1 also promotes cell migration and invasion.^108^ Knockdown of IP6K1 expression in cancer cells leads to a reduction in cell migration, invasion and anchorage-independent growth. *Ip6k1* knockout mice fed with the oral carcinogen 4NQO showed reduced progression from epithelial dysplasia

**Dysplasia**: Enlargement of an organ or a tissue due to excessive proliferation of cells of an abnormal type.

to invasive carcinoma in the upper aerodigestive tract as compared with their wild-type littermates, showing that IP6K1 is also required to promote cell invasion in vivo. This phenotype of resistance to aerodigestive tract carcinoma seen in *Ip6k1* knockout mice is in direct contrast to the outcome of 4NQO treatment observed in *Ip6k2* knockout mice (described earlier), which showed a higher incidence of carcinoma.^91^ These contrasting observations suggest that in aerodigestive tract epithelial cells, IP6K1 is responsible for promoting carcinogenesis, whereas the predominant function of IP6K2 is to prevent transformation by promoting apoptosis.

It was recently shown that the third IP_6_ kinase isoform, IP6K3, also influences the actin cytoskeleton. This study examined the effect of the loss of *Ip6k3* in specialised neurons in the cerebellum called Purkinje cells, which express high levels of IP6K3.^72^ Behavioural tests revealed that *Ip6k3* knockout mice manifest defects in motor learning and coordination. These mice showed abnormalities in synapse

**Synapse**: Junction between two nerve cells, which consists of a small gap across which impulses pass by diffusion of a neurotransmitter.

number and structure of Purkinje cells. The shape of Purkinje cell dendritic spines

**Dendritic spines**: Small protrusion from a neuron’s dendrite which receives signal from a single axon at the synapse.

is regulated by the arrangement and attachment of cytoskeletal elements including actin and actin-regulating proteins such as spectrins and adducins.^109^, ^110^ Spectrin is a structural protein that forms a mesh on the cytoplasmic face of the plasma membrane and adducin binds spectrin to promote its association with filamentous actin.^111^ IP6K3, but not IP6K1 or IP6K2, was shown to bind spectrin and adducin, and cells lacking IP6K3 showed reduced spectrin-adducin interaction.^72^ Catalytically active and inactive forms of IP6K3 can promote adducin binding to spectrin, providing another example of IP_6_ kinases functioning as structural scaffolds independent of their ability to synthesize PP-IPs.

**Cytoskeleton**: A network of protein filaments and tubules in the cytoplasm of cells, which gives them shape.

### Nucleus

The work of several laboratories has shed light on the importance of PP-IPs in the regulation of essential housekeeping functions in the nucleus, including the maintenance of genome integrity and regulation of transcription.

#### Genome Integrity

PP-IPs have been shown to participate in many processes that are responsible for maintaining the integrity of the eukaryotic genome. Telomeres are protein–DNA structures present at the ends of linear eukaryotic chromosomes to protect them against degradation. The repetitive DNA sequences present in telomeres shorten with each cell division and telomere shortening correlates with ageing. Two independent studies showed that PP-IPs play a role in maintaining the length of telomeres in budding yeast. Using yeast mutants deleted for various inositol phosphate biosynthetic enzymes, it was shown that that the loss of PP-IPs led to telomere lengthening, whereas their overproduction led to shortening of telomeres.^112^ Telomere lengthening in *kcs1*Δ yeast could be reversed by expression of active but not inactive Kcs1, indicating that PP-IP synthesis by this enzyme is necessary to maintain normal telomere length. In another study, it was observed that yeast lacking PP-IPs are resistant to treatment with wortmannin and caffeine, inhibitors of phosphoinositide 3-kinase (PI3K) and PI3K-related protein kinases, which are known regulators of telomere length.^113^ Yeast lacking Kcs1 showed longer than normal telomeres, but interestingly, yeast lacking the IP_6_ synthesizing enzyme Ipk1 showed shortening of telomeres. This was attributed to the high levels of inositol pyrophosphates PP-IP_4_ and [PP]_2_-IP_3_ synthesized from IP_5_ in these cells, so that the total PP-IP component of *ipk1*Δ yeast is actually higher than wild-type yeast. This data showed that any PP-IP can act to maintain telomere length, suggesting that perhaps protein(s) involved in telomere length maintenance are pyrophosphorylation targets of PP-IPs.

PP-IPs have been shown to participate in two key DNA repair pathways, homologous recombination (HR) mediated repair and nucleotide excision repair (NER). The yeast IP_6_ kinase Kcs1 (kinase C suppressor-1) was initially identified in a genetic screen for second site mutations that suppress the hyperrecombination phenotype observed in yeast carrying a mutant form of protein kinase C (Pkc).^114^ Subsequently, it was shown that the inositol pyrophosphate synthesis activity of Kcs1 is essential to support hyperrecombination in *pkc* mutant yeast.^115^ Our data revealed that the DNA recombination promoting function of PP-IPs is also conserved in mammalian cells.^116^ When MEFs were allowed to recover from DNA damage induced by the replication stress-inducer hydroxyurea or the radiomimetic antibiotic neocarzinostatin, cells lacking IP6K1 showed delayed entry into the next phase of the cell cycle, and ultimately underwent cell death. The DNA repair markers, γ-H2AX, BLM helicase, and Rad51 were recruited to the sites of DNA damage in *Ip6k1* ^−/−^ MEFs, suggesting that HR is initiated in these cells. However, these markers persisted for a longer time in *Ip6k1* ^−/−^ MEFs, and the DNA breaks were not repaired, indicating that repair is incomplete in these cells. Expression of active but not inactive IP6K1 could reverse this phenotype, showing that 5-IP_7_ is required to support HR in mammals. It was recently reported that IP6K1 also promotes NER in an enzyme activity dependent manner.^117^ IP6K1 was shown to interact with damage-specific DNA binding protein 1 (DDB1), which is part of the Cullin-RING ubiquitin ligase CRL4 complex, an E3 ubiquitin ligase that initiates NER.^118^ IP6K1 binding to CRL4 promotes its interaction with the COP9 signalosome (CSN) to keep the E3 ligase inactive. UV exposure leads to dissociation of DDB1 from IP6K1, allowing the synthesis of 5-IP_7_, which acts as a transducer for NER by promoting the dissociation of CRL4 from CSN.

#### Chromatin Remodelling

Two independent studies highlighted the role of PP-IPs in regulating epigenetic modifications in yeast and mammals. In budding yeast, PP-IPs were shown to play a critical role in regulating the environmental stress response. The yeast strain *kcs1*Δ*vip1*Δ, which is incapable of producing any PP-IPs, showed little to no transcriptional response to heat, osmotic, or oxidative stress.^61^ These cells displayed a decrease in stress dependent histone deacetylation brought about by the HDAC complex Rpd3L. A putative inositol phosphate binding site was identified on the catalytic subunit Rpd3, suggesting that PP-IP binding may directly activate Rpd3L to regulate the global transcription response to environmental stress. Another study identified that mouse IP6K1 interacts with the histone demethylase JMJD2C (Jumonji domain containing 2C).^119^ Cells lacking IP6K1 showed a global reduction in the levels of trimethylation on histone H3 lysine 9 (H3K9me3), and a concomitant increase in H3K9 acetylation. Overexpression of active but not inactive IP6K1 led to dissociation of JMJD2C from chromatin and a consequent increase in H3K9me3 levels. It was suggested that IP_7_ acts on one or more chromatin associated proteins to lower JMJD2C recruitment to chromatin.

#### Transcription Regulation

Studies in budding yeast have shown that PP-IPs control transcription to regulate major metabolic pathways. The Pho80–Pho85 cyclin-cyclin dependent kinase (CDK) complex is responsible for regulating the phosphate (Pi) responsive (PHO) pathway by phosphorylating the transcription factor Pho4 to promote its cytoplasmic accumulation. Under Pi starvation conditions, the CDK inhibitor Pho81 lowers the kinase activity of Pho80–Pho85, leading to dephosphorylation and nuclear translocation of Pho4 to trigger the transcription of PHO genes.^120^, ^121^ O’Shea and colleagues found that PP-IPs produced by Vip1 during phosphate starvation lowered Pho4 phosphorylation by the Pho80–Pho85–Pho81 complex.^57^ They further determined that 1-IP_7_ binding to Pho81 triggers a structural change that occludes the binding of Pho4 to the Pho85 kinase active site, but that 5-IP_7_ does not have this effect,^38^ highlighting how PP-IP binding-mediated regulation of protein function is specific to individual PP-IP isoforms. Another example of transcription regulation by 1-IP_7_ was seen in the mammalian innate immune response.^122^ 1-IP_7_ but not 5-IP_7_ increased phosphorylation and activation of the transcription factor IRF3, which is responsible for production of the cytokine IFNβ upon viral infection. Interestingly, a non-hydrolysable analogue of 1-IP_7_ could not recapitulate this effect, suggesting that 1-IP_7_-mediated specific pyrophosphorylation may be involved in this pathway. This study provides the only hint that PP-IP-mediated protein pyrophosphorylation may also be stereo-selective towards a particular isoform.

Yeast lacking Kcs1 exhibit inositol auxotrophy and decreased intracellular inositol levels.^123^ This is due to reduced transcription of the *INO1* gene which encodes *myo*-inositol-3-phosphate synthase, the enzyme that converts glucose-6-phosphate to inositol-3-phosphate, the rate limiting step of *de novo* inositol synthesis in eukaryotes. Inositol depletion in the growth medium led to increased Kcs1 protein levels, and PP-IPs synthesized by Kcs1 were found to be essential for the upregulation of *INO1* transcription under these conditions. As expression of mouse IP6K1 in *kcs1*Δ yeast rescued their inositol auxotrophy,^124^ it was expected that PP-IP dependent regulation of inositol synthesis would be conserved in mammals. However, MEFs lacking IP6K1 exhibited an unexpected increase in mammalian *Ino1* transcription, and a corresponding increase in inositol levels as compared with wild-type MEFs.^124^ This study further demonstrated that IP6K1 is localised to the nucleus by binding to the lipid phosphatidic acid, and that PP-IP synthesis by IP6K1 increases methylation of *Ino1* DNA to reduce its transcription. The phenomenon of *Ino1* transcription regulation by PP-IPs is a rare example of evolutionary divergence between yeast and mammals with regard to the influence of PP-IPs on a specific metabolic pathway.

Studies in our laboratory have shown that PP-IPs regulate rRNA transcription by RNA polymerase I in budding yeast.^45^ *kcs1*Δ yeast exhibit reduced protein synthesis due to a decrease in ribosome biogenesis, which in turn is attributed to a decrease in rRNA levels. Although there was no defect in the recruitment of RNA polymerase I on rDNA, the rate of transcription elongation was reduced in *kcs1*Δ as compared with wild-type yeast. We identified that 5-IP_7_ can pyrophosphorylate three subunits of the RNA polymerase I complex, and suggest that this modification may be essential to maintain normal transcription elongation by this polymerase. A recent study by the Fiedler group has shown that several potential IP_7_ pyrophosphorylation targets are nucleolar proteins associated with RNA polymerase I, suggesting that there may be additional proteins on which IP_7_ acts to regulate rRNA synthesis.^125^

## Perspective on the Future

Although PP-IPs were identified more than 20 years ago, there are still only a handful of researchers attempting to uncover the physiological functions of these unique molecules. The main reason for the lack of popularity faced by these molecules is that their study remains technically challenging for most cell biologists. There are no commercially available kits for the detection and measurement of PP-IPs, no fluorescently tagged or radiolabelled PP-IPs readily available for use in protein binding or pyrophosphorylation assays, and no easy methods to detect pyrophosphorylated proteins. Recent advances by the Fiedler, Jessen and Potter laboratories are likely to help in surmounting these challenges.^78^, ^79^, ^126^–^129^ Highly pure PP-IPs with β-phosphate moieties at specific carbon atoms have been synthesized by these groups, including non-hydrolysable analogues that are stable in cells and can bind target proteins but not pyrophosphorylate them.^47^, ^78^, ^130^ A system for intracellular delivery and photouncaging of chemically synthesized PP-IPs has recently been developed,^79^ which promises to open up new methods to study the functions of these molecules in different subcellular compartments. The use of IP_7_ as an affinity reagent revealed two different classes of interacting proteins, depending on the absence or presence of the divalent cation Mg^2+^ during the interaction, representing IP_7_ binding or pyrophosphorylation targets respectively.^125^ These latest advances promise a bright future for PP-IPs, with the hope that availability of new tools and information on novel PP-IP target proteins will draw new researchers into examining whether these versatile small molecules can regulate the protein or pathway of their interest.
